# Visualization of cell-cycle modification by ionizing irradiation in single HeLa cells using fluorescent ubiquitination-based cell-cycle indicator

**DOI:** 10.1093/jrr/rrt159

**Published:** 2014-03

**Authors:** Kiichi Kaminaga, Yuka Sakamoto, Yukiko Kanari, Miho Noguchi, Akinari Yokoya

**Affiliations:** 1 Ibaraki University, Japan; 2 JAEA, Japan

**Keywords:** cell cycle, FUCCI, time-lapse imaging

## Abstract

It has been known that cell cycle is retarded or arrested when the cells are exposed to ionizing radiation. The cell-cycle modifications are thought to be controlled by check point mechanisms to ensure the time for DNA repair. Linear energy transfer (LET) dependence of cell-cycle modifications, however, has not been fully revealed. Considerably less is known about detailed cell-cycle arrest for a single-cell level after exposure. Our purpose is to explore high LET radiation effects on the mammalian cell cycle. To examine high LET radiation effects on mammalian cell cycle, it would be essential to track single cells as live cell images observed by time-lapse imaging technique. HeLa cells expressing fluorescent ubiquitination-based cell-cycle indicator (FUCCI) are one of the useful model cell lines to visualize cell cycle because their nuclei show different colors; orange indicating G1 (Cdt1 expression); green indicating S/G2 (Geminin expression) [ 1]. In order to establish a novel assay system to study cell-cycle modification by high LET irradiation such as ion beams, we have developed time-lapse protocol for the HeLa-FUCCI cells irradiated.

As a preliminary experiment using conventional X-rays instead of high LET ion beams, we observed the cell cycles of the irradiated HeLa cells. Figure 1 shows a typical time-lapse dynamics of unirradiated cells acquired for 48 h. We establish a new method to decide the time of one cell cycle as shown in Fig. 1. We obtained an evidence that the irradiated (5 Gy) cells show prolonged cell-cycle period when compared with that of control cells (Table 1). We also revealed that the delay is mainly caused in the G2 (Geminin expressing) phase. These results suggest that S/G2, G2/M or M checkpoint mechanism regulate the cell cycle in the irradiated HeLa-FUCCI cells. In conclusion, single FUCCI cell exposure and live cell imaging are a powerful method to trace the single-cell effect of high LET irradiation on the cell cycle in future.

**Fig. 1.**
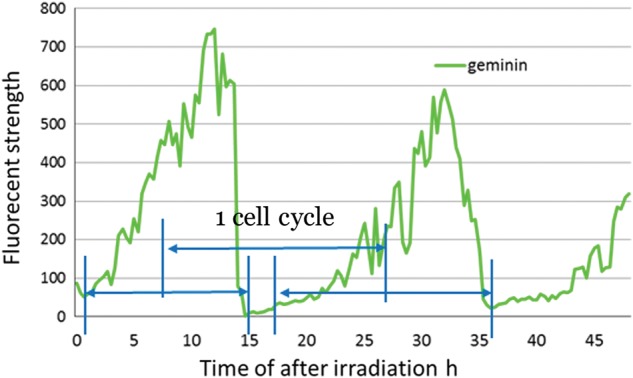
Typical time-lapse dynamics of an unirradiated HeLa-FUCCI cell monitored by geminin fluorescent intensity of the cell nucleus.

**Table 1.** Distribution of the number of cells showing a specific cell cycle period.

Cell cycle period, h	Number of cells	
	Control (unirradiated)	5 Gy irradiated
>14	6	2
14–16	8	3
16–18	12	9
18–20	22	16
20–22	15	8
22–24	5	8
24–26	5	7
26–28	1	6
28–30	1	0
30 <	0	8
Total	75	67

Typical time-lapse dynamics of an unirradiated HeLa-FUCCI cell monitored by geminin fluorescent intensity of the cell nucleus.

Distribution of the number of cells showing a specific cell cycle period.

## References

[1] Sakaue-Sawano A (2008). Cell.

